# Application of three-step laparoscopic cholecystectomy combined with indocyanine green fluorescence imaging in acute difficult cholecystitis

**DOI:** 10.3389/fmed.2026.1878390

**Published:** 2026-07-23

**Authors:** Jian-Yuan Lin, Shao-Xiong Zheng, Feng Lin, Chun-Cheng Li, Jian-Ping Wu, De-Xian Xiao, Deng-Fang Guo, Chun Zhang

**Affiliations:** 1 Department of General Surgery, Mindong Hospital Affiliated to Fujian Medical University, Ningde, Fujian, China; 2 General Surgery Department, Shouning County Hospital, Ningde, Fujian, China; 3 General Surgery Department, Zherong County Hospital, Ningde, Fujian, China

**Keywords:** complicated acute cholecystitis, fluorescence imaging, indocyanine green, surgical complications, three-step laparoscopic cholecystectomy

## Abstract

**Introduction:**

This study aimed to evaluate the clinical value of three-step laparoscopic cholecystectomy combined with indocyanine green (ICG)-guided fluorescence imaging in patients with complicated acute cholecystitis.

**Methods:**

We retrospectively reviewed the clinical data of 115 patients with complicated acute cholecystitis treated at three hospitals in Ningde between January 2022 and June 2024. The patients were assigned to either a conventional laparoscopic surgery group (60 cases) or a three-step laparoscopic cholecystectomy combined with ICG fluorescence imaging group (55 cases). Operative time, intraoperative blood loss, postoperative recovery indicators, and complication rates were compared between the two groups.

**Results:**

The combined group showed significantly better outcomes than the conventional group with respect to operative time, intraoperative blood loss, time to postoperative flatus, drainage duration, and length of hospital stay (*P* < 0.001). The complication rate in the combined surgery group was 7.2%, which was significantly lower than the 20.0% observed in the conventional surgery group (*P* < 0.05).

**Conclusion:**

Three-step laparoscopic cholecystectomy combined with ICG fluorescence imaging can clearly delineate biliary anatomy, enhance surgical exposure; it reduces the risk of postoperative complications and bile duct injury, improves surgical safety in complicated acute cholecystitis, accelerates postoperative recovery, and has considerable value for clinical application.

## 1 Introduction

Acute cholecystitis is one of the common acute abdominal conditions in general surgery. With the deepening of population aging, its incidence continues to rise. Laparoscopic cholecystectomy has been widely adopted in clinical practices due to its minimal invasiveness, mild postoperative pain, and rapid postoperative recovery, and has become the gold-standard procedure for benign gallbladder lesions (1). However, the incidence of iatrogenic bile duct injury (BDI) caused by this procedure remains approximately 0.3%–0.5% (2). Meanwhile, influenced by factors such as recurrent acute inflammation, gallbladder atrophy, cystic duct stones, and biliary anatomical abnormalities, the gallbladder and adjacent tissues are severely distorted with dense adhesions, leading to obscured anatomical planes, with anatomical layers becoming blurred and fused. Approximately 10%–15% of acute cholecystitis cases are classified as complicated acute cholecystitis (3). Severe anatomical distortion in complicated gallbladders impedes conventional laparoscopic dissection of Calot’s triangle and adequate isolation of the cystic duct and artery, making it difficult to establish the critical view of safety (CVS), thus further increasing the risk of bile duct injury (BDI) and vascular injury (4). Vascular injury can cause uncontrollable bleeding, while BDI may lead to a series of serious consequences such as bile leakage, bile peritonitis, biliary stricture, biliary cirrhosis, and even liver failure. Such adverse events constitute the primary indications for intraoperative open conversion and reoperation, directly threatening patient safety and quality of life and increasing perioperative morbidity (2). Precisely because of this, how to safely perform laparoscopic cholecystectomy for complicated acute cholecystitis has become a realistic and urgent clinical challenge in clinical practice.

Indocyanine green (ICG) is a safe, non-toxic near-infrared fluorescent dye that is nearly fully hepatocellularly absorbed and subsequently excreted via the biliary tree. Owing to these properties, ICG fluorescence imaging has been increasingly used in hepatobiliary surgery (5). Several studies have shown that ICG enables visualization of biliary anatomy during complicated acute cholecystitis, provides real-time intraoperative biliary imaging, and effectively reduces the risk of BDI (6–12). Laparoscopic three-step cholecystectomy is a modified form of laparoscopic subtotal cholecystectomy recently reported by our team; it combines the advantages of retrograde cholecystectomy and subtotal cholecystectomy (13). Compared with conventional laparoscopic cholecystectomy, this approach may further reduce the likelihood of BDI and vascular injury in complicated acute cholecystitis, thereby improving safety and reducing complications (4). By evaluating three-step laparoscopic cholecystectomy combined with ICG fluorescence imaging in complicated acute cholecystitis, this study aimed to explore a new strategy for improving surgical safety and reducing operative complications.

## 2 Materials and methods

### 2.1 General information

This retrospective study included clinical data from 115 patients with complicated acute cholecystitis who were admitted to three hospitals in Ningde from January 2022 to June 2024. The patients were divided into a conventional laparoscopic surgery group (60 cases) and a three-step cholecystectomy combined with ICG fluorescence imaging group (55 cases). All patients signed written informed consent. The study met ethical requirements, and all clinical data were collected solely for clinical research purposes.

Inclusion Criteria

The inclusion criteria were as follows: (1) an acute attack of cholecystitis or acute exacerbation of chronic cholecystitis; (2) an enlarged or atrophic gallbladder, or impacted stones in the cystic duct or gallbladder neck; and (3) preoperative imaging suggesting cystic duct variation or intraoperative findings of marked inflammatory edema and dense adhesions in Calot’s triangle, making the bile ducts and blood vessels difficult to distinguish.

Exclusion Criteria

The exclusion criteria were as follows: (1) poor general condition or severe cardiopulmonary dysfunction leading to inability to tolerate surgery; (2) concomitant acute pancreatitis, common bile duct stones, or acute cholangitis; (3) suspected gallbladder malignancy before or during surgery; (4) a history of open surgery; (5) severe decompensated cirrhosis with ascites or hematological disease related to coagulation dysfunction; (6) loss to postoperative follow-up; and (7) allergy to ICG.

### 2.2 Surgical methods

The specifications and brands of the instruments used in the conventional surgery group and in the three-step cholecystectomy combined with ICG fluorescence imaging group were consistent, and the same fluorescence laparoscopic equipment was used in both groups.

**FIGURE 1:**
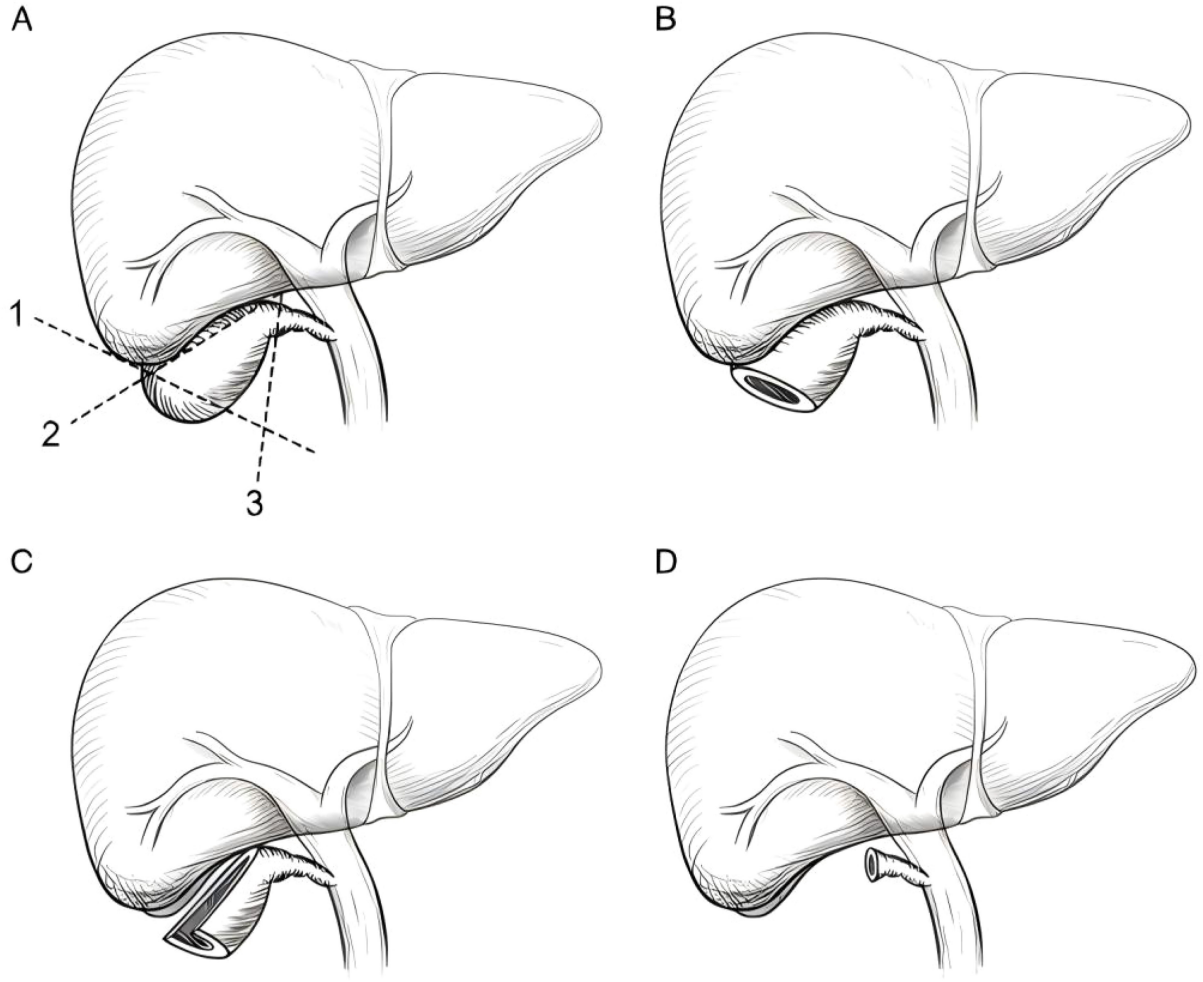
Three-step laparoscopic cholecystectomy. **(A)** General schematic diagram of 3-step operation. **(B)** Resection of the bottom of the gallbladder. **(C)** Preserve part of the gallbladder wall on the liver surface and split the gallbladder up to the neck of the gallbladder. **(D)** Residual cervical duct of the gallbladder.

**FIGURE 2:**
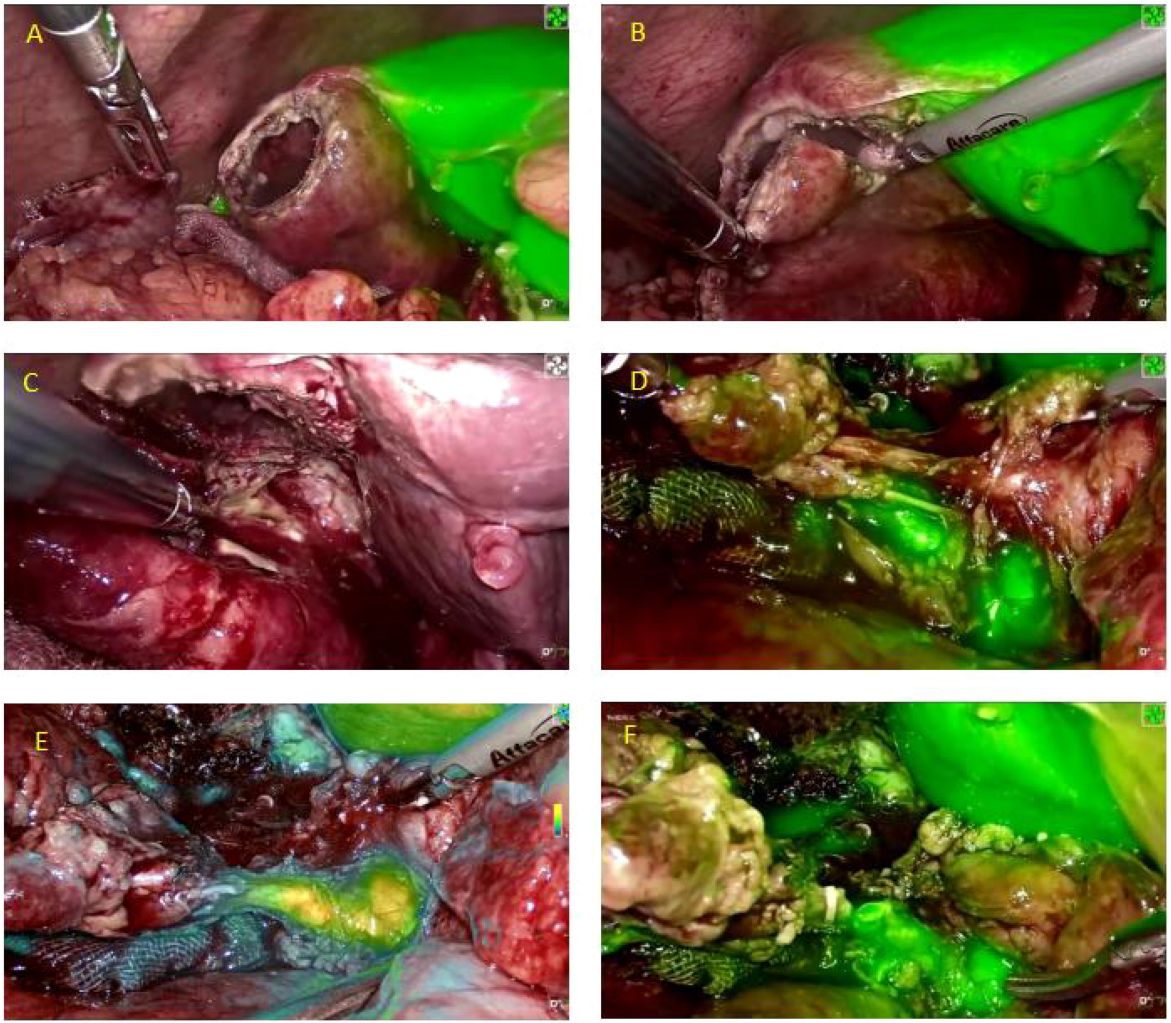
**(A)** Resection of the bottom of the gallbladder. **(B)** Preserve part of the gallbladder wall on the liver surface and split the gallbladder up to the neck of the gallbladder. **(C)** Expose the area of the Calot’s triangle. **(D,E)** Under ICG fluorescence imaging, the cystic duct and artery were precisely dissected. **(F)** Confirm that there are no residual stones in the bile duct. Ensure that the bile duct is unobstructed and that the bile flows smoothly. Then, close or suture the bile duct.

Combined surgery group: Peripheral intravenous ICG was administered 30 min preoperatively at a dose of 0.05 mg/kg (ICG working concentration: 0.25 mg/mL). After peritoneal access was established, the laparoscopic fluorescence mode was activated to preliminarily visualize the biliary structures. Laparoscopic three-step cholecystectomy was performed according to the procedure previously reported by our team (Figure 1) (13). The ICG fluorescence-guided steps were as follows. Step 1: The gallbladder was decompressed via bile aspiration, and the fundus was resected using an ultrasonic scalpel to retrieve intraluminal gallstones (Figure 2A). Step 2: The gallbladder wall adherent to the hepatic surface was preserved, and the gallbladder was dissected toward the neck (Figure 2B). Step 3: Calot’s triangle was dissected, followed by transection of the gallbladder at the neck (Figure 2C). Under ICG fluorescence imaging, the cystic duct and cystic artery were carefully dissected (Figures 2D, E). After the course of the cystic duct was confirmed, dissection was performed as close as possible to the cystic duct wall, and the absence of residual stones within the cystic duct was further verified. After free flow of bile from the gallbladder and cystic duct was observed, the cystic duct was clipped or sutured (Figure 2F). After the procedure, fluorescence mode was used again to observe the biliary tract and check for bile leakage and residual stones. Hemostasis was achieved in the operative field, the area was rinsed with saline, and a drainage tube was placed. All operations were completed by a single senior surgeon to eliminate inter-surgeon technical bias.

Conventional surgery group: A standard four-port abdominal approach was used. The gallbladder was dissected under white-light mode; the cystic duct and cystic artery were isolated; and conventional antegrade or retrograde cholecystectomy was performed.

### 2.3 Observation indicators

General patient information, including sex, age, and body weight, was recorded. Operative time, intraoperative blood loss, time to postoperative flatus, postoperative drainage duration, postoperative length of stay, and intraoperative or postoperative complications were observed and compared between the two groups. Complications included massive intraoperative hemorrhage, bile duct injury, bile leakage, secondary postoperative common bile duct stones, infected subphrenic fluid collection, and postoperative pulmonary infection.

### 2.4 Statistical methods

All statistical analyses were performed using SPSS 20.0 software. Continuous variables were presented as mean ± standard deviation (SD) and compared with independent-sample *t*-tests. Count data were expressed as rates (%) and analyzed using χ^2^ tests. *P*-value < 0.05 was considered statistically significant.

## 3 Results

### 3.1 Comparison of intraoperative and postoperative indicators between the two groups

Intraoperative and postoperative parameters, including operative duration, estimated blood loss, time to first flatus, drainage duration and hospital stay, were all significantly superior in the combined group (*P* < 0.05). The specific data are shown in Table 1.

**TABLE 1:** Comparison of intraoperative and postoperative conditions between the two groups of patients (x̄ ± s).

Group	Operation time (min)	Intraoperative bleeding volume (ml)	Postoperative exhaust time (h)	Postoperative drainage time (d)	Postoperative hospital stay (d)
Combined group (*n* = 55)	84.7 ± 10.1	37.8 ± 10.2	23.5 ± 3.3	2.5 ± 0.8	5.9 ± 0.8
Conventional group (*n* = 60)	118.4 ± 14.4	68.9 ± 13.8	32.0 ± 4.6	3.5 ± 0.8	7.6 ± 1.0
*T*-value	14.612	13.773	11.379	6.323	10.081
*P*-value	<0.001	<0.001	<0.001	<0.001	<0.001

### 3.2 Comparison of postoperative complication rates between the two groups

The overall incidence of postoperative complications in the combined surgery group was 7.2%, which was significantly lower than the 20% observed in the conventional surgery group; the difference was statistically significant (*P* < 0.05). The specific data are shown in Table 2. All patients recovered and were discharged after conservative treatment, and no reoperations were required in either group.

**TABLE 2:** Comparison of complication occurrence between the two groups of patients (%).

Group	Intraoperative massive hemorrhage	Bile duct injury	Bile leakage	Secondary common bile duct stones	Subphrenic effusion	Pulmonary infection	Total
Combined group (*n* = 55)	0 (0)	0 (0)	1 (1.8)	0 (0)	1 (1.8)	2 (3.6)	4 (7.2)
Conventional group (*n* = 60)	0 (0)	0 (0)	3 (5.0)	1 (1.7)	3 (5.0)	5 (8.3)	12 (20.0)
χ^2^ value							3.881
*P*-value							0.049

## 4 Discussion

Because of the high risk of bile duct and vascular injury, complicated acute cholecystitis was previously considered a contraindication to laparoscopic cholecystectomy (14). Although percutaneous gallbladder drainage is widely used in the management of complicated acute cholecystitis because of its safety and effectiveness (15), it cannot resolve stones within the gallbladder. Additional surgery is often needed to address the underlying cause, which not only prolongs the treatment course but also imposes physical and psychological burdens on patients because of the discomfort associated with long-term catheterization (15, 16). Therefore, it is not the optimal treatment for complicated acute cholecystitis (15). Meanwhile, with the widespread acceptance of modern minimally invasive concepts, traditional open surgery no longer meets patient expectations (17). Accordingly, improving the safety of laparoscopic cholecystectomy for complicated acute cholecystitis and lowering postoperative complication rates have become urgent clinical priorities (18, 19). In recent years, technological progress has promoted upgrades in surgical equipment, including laparoscopic systems, electrosurgical devices, and suture materials, providing strong support for the improvement of surgeons’ technical skills (17). At the same time, innovations in minimally invasive concepts and techniques, including the CVS, the principle of “prioritizing gallbladder injury over liver injury” during gallbladder dissection, retrograde cholecystectomy, and laparoscopic subtotal cholecystectomy (19), have supported the safe implementation of LC (20, 21). However, important limitations remain when managing the anatomy of complicated acute cholecystitis.

The main challenges in laparoscopic surgery for complicated acute cholecystitis are the difficulty of identifying anatomical structures and the limited operative space (14, 15). Recurrent inflammatory episodes induce dense adhesions between the gallbladder and surrounding liver, bowel and omental tissues, which severely impairs intraoperative recognition of anatomical landmarks. Consequently, clear dissection of a difficult gallbladder to obtain the CVS remains highly challenging. Forceful dissection under restricted visualization can easily damage surrounding organs, especially in Calot’s triangle, leading to serious complications such as bleeding, bile leakage, and biliary stricture (20). In this study, the combined surgery group was superior to the conventional group in operative time, intraoperative blood loss, postoperative intestinal function recovery time, postoperative drainage duration, postoperative length of stay, and postoperative complication rate. These findings confirm that three-step cholecystectomy combined with ICG fluorescence imaging can significantly improve the safety of laparoscopic surgery for complicated acute cholecystitis and reduce the risk of complications. The three-step cholecystectomy technique first reduces gallbladder tension, allowing better intraoperative control of the gallbladder. Removal of the gallbladder fundus permits direct observation of the gallbladder cavity and simultaneous stone extraction, thereby expanding the operative field and avoiding injuries caused by blind dissection. Preserving the gallbladder wall on the liver side helps prevent intraoperative liver injury and postoperative wound oozing. Leaving Calot’s triangle until the final step avoids blind manipulation in the area of severe inflammation and markedly reduces the risk of BDI. Therefore, three-step cholecystectomy provides a strategic safety zone for managing difficult gallbladder anatomy. ICG fluorescence imaging provides real-time dynamic visualization of the cystic duct, common hepatic duct, common bile duct, and variant biliary branches, offering the surgeon a precisely guided “navigation system” during adhesiolysis and management of Calot’s triangle. The combination of the three-step strategy and real-time ICG fluorescence navigation reduces dissection difficulty and minimizes iatrogenic injury to the liver, bowel, biliary tree and pericholecystic vessels. In addition, real-time fluorescence guidance allows the cystic duct to be managed more appropriately, reducing postoperative bile leakage caused by insufficient closure of the cystic duct stump and lowering the likelihood of cystic duct stones entering the common bile duct during manipulation.

## 5 Conclusion

In summary, the three-step cholecystectomy creates sufficient surgical space, whereas ICG fluorescence imaging offers real-time intraoperative anatomical navigation to facilitate structure recognition. The combined strategy standardizes and visualizes complex biliary dissection procedures, shortens the operative process, reduces the rate of conversion to open surgery, and decreases intraoperative and postoperative complications. This approach is worthy of further clinical promotion.

## 6 Limitations

This study has several limitations, including a small sample size and incomplete observation indicators. Therefore, future research should focus on expanding the sample size, improving observation indicators, delayed follow-up time, and further exploration of the application value of the three-step laparoscopic cholecystectomy combined with ICG fluorescence imaging technology in acute difficult cholecystitis,in order to strengthen guidance for clinical practice.

## Data Availability

The original contributions presented in this study are included in this article/supplementary material, further inquiries can be directed to the corresponding authors.
